# AI-Driven Predictions of Readmission and Mortality for Improved Discharge Decisions in Critical Care: A Retrospective Study

**DOI:** 10.3390/diagnostics16060874

**Published:** 2026-03-16

**Authors:** Yeonjeong Heo, Minkyu Kim, Seon-Sook Han, Tae-Hoon Kim, Jeongwon Heo, Dohyun Kim, Woo Jin Kim, Seung-Joon Lee, Oh Beom Kwon, Yoon Kim, Hyun-Soo Choi, Da Hye Moon

**Affiliations:** 1 Department of Internal Medicine, Kangwon National University, Chuncheon 24341, Republic of Korea; yonjong1954@naver.com (Y.H.); ssunimd@kangwon.ac.kr (S.-S.H.); pulmo2@kangwon.ac.kr (W.J.K.); medfman@kangwon.ac.kr (S.-J.L.); obkwon@kangwon.ac.kr (O.B.K.); 2 Department of Research & Development, Ziovision Co., Ltd., Chuncheon 24341, Republic of Korea; gim4855744@gmail.com (M.K.); dohyeon.kim@ziovision.co.kr (D.K.); yooni@ziovision.co.kr (Y.K.); 3 Institute of Medical Science, School of Medicine, Kangwon National University, Chuncheon 24341, Republic of Korea; blessing0104@naver.com; 4 Department of Pulmonology, Chinjujeil Hospital, Jinju 52709, Republic of Korea; doctorhjw@naver.com; 5 Department of Computer Science and Engineering, Kangwon National University, Chuncheon 24341, Republic of Korea; 6 Department of Computer Science and Engineering, Seoul National University of Science and Technology, Seoul 01811, Republic of Korea

**Keywords:** artificial intelligence, discharge, readmission, mortality

## Abstract

**Background/Objectives**: The transition from the intensive care unit (ICU) to the hospital ward is a critical high-risk period for patients. Early ICU discharge reduces costs and frees up ICU resources but can lead to readmission or unexpected death if patients are discharged prematurely. Despite the availability of risk stratification tools such as the Stability and Workload Index for Transfer (SWIFT) score, predicting ICU readmission remains challenging and inconsistent. However, artificial intelligence (AI) and machine learning (ML) techniques have recently shown promise in improving clinical decision support systems, particularly in the ICU. This study aimed to identify the risk factors and assess the performance of AI models in predicting readmission or death within seven days of ICU discharge using the MIMIC-IV (between 2008 and 2019) and Kangwon National University Hospital (KNUH, between 1 January 2016 and 28 February 2023) databases. **Methods**: This retrospective cohort study utilized the MIMIC-IV database for model training and internal validation and the KNUH database for external validation. Various machine learning and deep learning models have been developed to predict ICU readmission or death within seven days of discharge. The performance of the primary model, GRU-D++, was compared to the SWIFT score. Statistical analysis focused on the area under the receiver operating characteristic curve (AUROC) data to evaluate model accuracy. **Results**: The GRU-D++ model outperformed the SWIFT score, achieving AUROC of 0.802 and 0.756 for internal and external validations, respectively. Both datasets demonstrated that the GRU-D++ model provided better predictive performance for ICU readmission or death within seven days than the traditional SWIFT score. **Conclusions**: Our findings suggest that the GRU-D++ deep learning model is a valuable tool for the early detection of patient deterioration after ICU discharge, potentially aiding the prevention of ICU readmission. This study highlights the potential of AI to improve clinical decision-making in intensive care settings.

## 1. Introduction

Discharge of patients from the intensive care unit (ICU) to the hospital ward is one of the most challenging and high-risk transitions of care during which critically ill patients change their physicians and nurses [1]. Early discharge from the ICU is desirable because it shortens ICU time and reduces care costs. However, it can also increase the likelihood of ICU readmission and unanticipated post-discharge death if patients are discharged before they become stable [2]. In contrast, prolonged ICU stays are costly and stressful for patients and families, reduce the number of beds available for other patients, and increase the risk of iatrogenic and nosocomial complications [3]. The clinical burden of ICU readmission remains a global challenge. Recent studies report a mean ICU readmission rate of 7%, with a range between 4% and 14% [4]. Specifically, it has been observed that 8.6% of patients require readmission during their hospital stay, with unplanned readmission rates escalating from 1.9% within 24 h to 4.5% within 72 h after discharge [5]. These statistics underscore the necessity for reliable predictive tools to mitigate the risk of post-ICU clinical deterioration.

Determining when patients are ready for ICU discharge is a daily challenge for physicians. Traditionally, this determination has depended on the clinical judgment of the physician discharging the patient from the ICU and the physician admitting the patient to the hospital ward. Nevertheless, there is no consensus on an ICU discharge risk-stratification tool. In the ICU, several tools, such as the Acute Physiology and Chronic Health Evaluation (APACHE) score [6] and the Glasgow Coma Scale (GCS) [7], have been used to evaluate patients’ conditions. Physicians often rely on these scores to make discharge decisions. However, these scores can be subjectively interpreted because of the nature of some variables [8].

Consequently, they may not consistently and accurately reflect the patient’s status. The Stability and Workload Index for Transfer (SWIFT) score was developed to overcome this limitation as a validated numerical tool designed to measure a patient’s safety and suitability for discharge from the ICU. Developed by Gajic et al. [9] in 2008, the SWIFT score predicts unplanned readmission. Logistic regression was used to analyze factors such as patient location before ICU admission, total length of ICU stay, last PaO2/FiO2 ratio, most recent GCS score, and most recent PaCO_2_ value. Nevertheless, although the SWIFT criterion is the only validated risk score for ICU discharge, it performs poorly and inconsistently upon implementation [10,11]. As noted in prior literature (e.g., Shi et al. [10]), the SWIFT score often demonstrates suboptimal performance, with AUROC values as low as 0.51 in external validation. As demonstrated in the study by Rosa et al. [11], the SWIFT score often exhibits low discriminative power, yielding an AUROC of only 0.58 in prospective cohorts. These limitations justify the development of more advanced AI-based models to improve the safety of ICU discharge transitions.

Risk stratification for patients discharged from the ICU is a complex process with many potential challenges [12]. Despite the critical need for accurate decision-making, few effective tools are currently available to help clinicians determine which patients are truly ready for discharge. Consequently, there has been increased use of machine learning (ML) and deep learning (DL) techniques for predictive modeling using routinely collected hospital data. Artificial intelligence (AI) models continue to evolve into clinical decision support systems [13], and these technologies have aided clinicians in various labor-intensive tasks such as rapid diagnosis, prediction of patient outcomes, risk stratification, optimizing resource allocation, and continuous patient monitoring. Thus, the role of AI in emergency and intensive care settings has recently garnered significant interest [14]. One notable resource contributing to this progress is the Medical Information Market for Intensive Care IV (MIMIC-IV) database [15], which offers a rich array of patient data in the ICU setting. Furthermore, the recent increase in access to public databases containing anonymized health records, particularly in intensive care units, has presented an opportunity to use machine learning to revolutionize clinical procedures [16].

In this study, we aimed to identify the risk factors associated with readmission or death within 7 days of discharge from the ICU and evaluate the performance of various AI models in predicting these outcomes. The 7-day readmission window was adopted as a clinical quality metric based on Hosein et al.’s meta-analysis, which identifies this interval as a consistent benchmark for assessing ICU-to-ward transitions. This timeframe provides a pooled cumulative incidence (4–6%) suitable for risk stratification and literature-based benchmarking. Consequently, our model utilizes this period to identify patients at high risk of deterioration during the critical post-discharge phase. To achieve this, we utilized patient data from the MIMIC-IV database for model training and internal validation, while external validation was conducted using data from Kangwon National University Hospital (KNUH). Additionally, we compared the performance of our deep learning model with that of the traditional ICU discharge assessment tool, the SWIFT scoring system.

## 2. Materials and Methods

### 2.1. Study Design and Population

In this study, we utilized the MIMIC-IV version 2.2 and KNUH datasets from the Republic of Korea. MIMIC-IV is a publicly available database containing patient information for those admitted between 2008 and 2019 at a Medical Center in the USA, comprising 73,181 ICU stays. We also used real-world datasets from an ICU in Korea between 1 January 2016, and 28 February 2023, during which 9812 ICU stays were identified. However, institutional ICU admission and discharge protocols remained standardized throughout this duration. This consistency ensured that the predictive variables were maintained within a stable clinical context without significant shifts in treatment standards.

Patients from the MIMIC-IV database were used for model training and internal validation, whereas those from the KNUH database were used for external validation. Initially, we screened 73,181 ICU stays from MIMIC-IV and 9812 from KNUH for patients aged ≥18 years. Stays were excluded if they met the following criteria: (1) ICU length of stay < 24 h, (2) mortality during the ICU stay, or (3) transfer to other specialized units or the operating room rather than a general ward.

**Figure 1:**
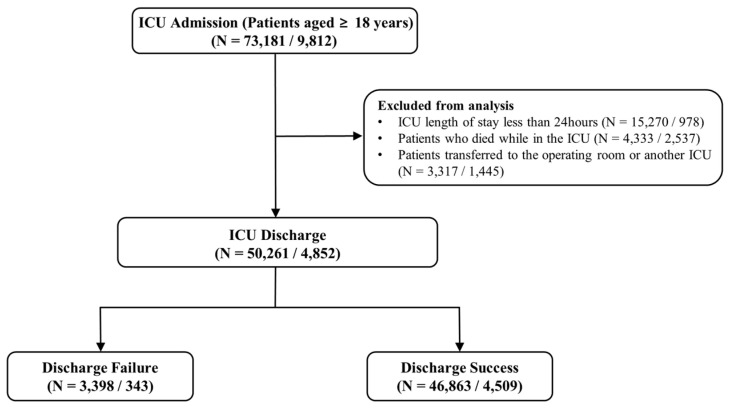
Flow diagram of MIMIC-IV and KNUH datasets (*N* = MIMIC-IV/KNUH). Abbreviations: ICU, Intensive care unit; MIMIC, Medical Information Mart for Intensive Care; KNUH, Kangwon National University Hospital.

The number of ICU stays from the MIMIC-IV included in this study was 50,261. Figure 1 illustrates the cohort selection process. The KNUH dataset contains 4852 ICU stays.

#### Ethics Statement

This study was approved by the Institutional Review Board of Kangwon National University Hospital (KNUH-2023-12-008-001, 5 June 2024). All the procedures were conducted strictly in accordance with the approved protocol, adhering to standard regulation and the principles of Declaration of Helsinki. De-identified electronic medical record data from KNUH were used in the study, and as a result, the IRB of KNUH waived the requirement for informed consent.

### 2.2. Data Pre-Processing

**Figure 2:**
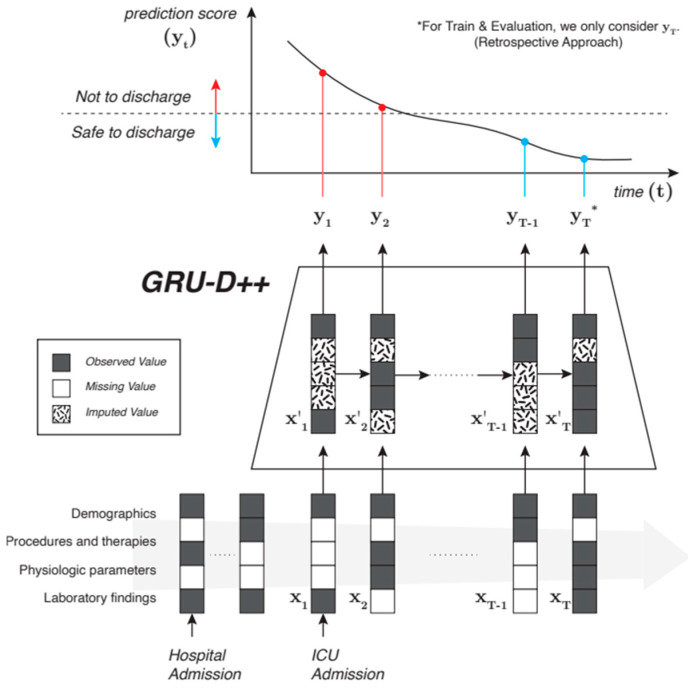
Overview of the study design and predictive model. Abbreviations: ICU, Intensive care unit; GRU, Gated Recurrent Unit.

ICU readmission was defined as a transfer from the ICU to the general ward and back to the ICU during the same hospital stay. The primary outcome of this study was readmission to the ICU or death within seven days of discharge from the ICU to the ward (Figure 2). This study focused on predicting the likelihood of patient death or readmission to the ICU within 7 days following discharge from the ICU. The input data for this prediction includes variables (denoted as x_t_) that may have missing values. To address this, the study employs a model called GRU-D++, which can automatically impute these missing values. For the training and evaluation phases, the model uses data from the final moments of the patient’s ICU stay, reflecting a retrospective approach. However, in a real-world setting, GRU-D++ is sufficiently flexible to make predictions at any time point in the patient’s timeline, allowing for prospective, continuous risk assessment after ICU discharge. Adult patients aged 18 years or older who were admitted to the ICU for more than 24 h were included. We also included patients in all the ICU and disease groups. Patients who died during ICU admission were excluded from this study. And readmissions were categorized into planned and unplanned. Planned surgical readmissions included scheduled ICU returns for staged post-operative care or planned interventions. Unplanned readmissions were defined as emergency ICU returns due to unanticipated clinical worsening. To evaluate discharge safety, only unplanned readmissions were included in the primary endpoint analysis; conversely, planned surgical readmissions were not considered readmissions but were modeled as one continuous ICU stay.

### 2.3. Data Collection

We initially collected 48 clinical variables based on previous studies [10,17]. All variables were collected at 1-day intervals and averaged when multiple measurements were taken within a day. While daily averaging was employed to mitigate the impact of transient physiological noise and ensure model stability, we acknowledge that this may overlook short-term variability. However, the GRU-D++ architecture’s decay mechanism partially compensates for this by focusing on longitudinal trends, which are highly relevant for predicting mid-term outcomes such as 7-day readmission. If there was at least one instance of missing data per day, it was classified as missing values. Owing to the high proportion of missing values in some of these variables, we selected only 26 variables, each with less than a 50% missing value rate, for our experiments. Variables exceeding this 50% threshold were excluded to ensure the robustness of the predictive model and to prevent potential bias stemming from excessive data imputation. This rigorous thresholding process resulted in a final set of 26 variables that were consistently available across both the development (MIMIC-IV) and external validation (KNUH) cohorts, thereby enhancing the model’s cross-institutional generalizability and ensuring its reliability in diverse clinical settings. Table 1 shows the selected 26 variables, including patient demographics, vital signs, laboratory findings, and treatment events. Before inputting the variables into an AI model, we normalized the numerical variables to have zero mean and unit variance. The missing rates of all variables initially collected are presented in Supplementary Table S1.

### 2.4. Statistical Analysis

Table 1 presents the statistics for all variables and the *p* values comparing the discharge success and failure groups. For the numerical variables, we report the mean and standard deviation, whereas, for the binary variables, we provide the count and proportion of positive values. We performed three normality tests: the Kolmogorov–Smirnov, Normal, and Jarque–Bera tests. The results from all tests indicated that none of the numerical variables followed a normal distribution, both in raw and log-transformed forms (*p* < 0.001). The normal distributions of the 19 variables with *p* < 0.001 in both the MIMIC and KNUH datasets are presented in Supplementary Figures S1 and S2. Consequently, we applied the Mann–Whitney U test to calculate the *p* values for the numerical variables. For binary variables (sex, HFNC, ventilator, dialysis, vasopressor, sedation, and transfusion), we used Fisher’s exact test to obtain *p* values.

### 2.5. Deep Learning Model GRU-D++ for Predictive Systems

Real-world patient information is continuously collected and can be viewed as time series data. However, most studies converted patient information into a simple tabular form, neglecting the historical context. Additionally, patient information stored in electronic health records (EHR) often contains missing values. Existing studies typically impute these missing values using simple methods, such as forward fill or mean fill, which do not consider contextual information (i.e., patient history). To address these problems, we proposed an advanced DL model denoted as a Gated Recurrent Unit with Decay++ (GRU-D++). The GRU-D++ model demonstrated superior performance in the early prediction of pressure ulcers [18]. Because conventional ML models cannot directly handle missing values, we initially imputed missing values using the last observed value of the variables (i.e., forward filling), and the remaining missing values were then imputed with the global mean of the variables (i.e., mean filling). The GRU-D++ models used in this study could directly handle missing values; thus, these imputations were not applied when using these models.

To evaluate the effectiveness of GRU-D++, we compared our system to nine widely used ML and advanced DL models. Notably, methods such as logistic regression, decision trees, random forests, and extreme gradient boosting [19] have been widely used in previous studies. However, these models cannot handle time-series data, making them unsuitable for the real-time data used in our study. Furthermore, recurrent neural networks (RNNs), GRU [20], and long short-term memory models (LSTMs) [21] are DL models designed for time-series analysis and can process real-time data. However, they do not effectively manage missing values, which are problematic in real-world scenarios. GRU-D [22] was proposed to address this issue as a GRU-based model that directly handles missing values. Existing GRU-D integrates temporal decay within its hidden states in conjunction with an automated imputation mechanism to handle missing data. Consequently, GRU-D exhibits superior predictive accuracy compared to the standard GRU architecture when applied to incomplete datasets. A primary limitation of GRU-D, however, is the assumption that variables at the initial time step are fully observed. GRU-D++ mitigates this constraint by refining the imputation mechanism to accommodate missing values in instances where no historical observations exist. The details of GRU-D++ are described in Appendix A. Additionally, we compared our proposed system with an existing clinical score for readmission and the SWIFT score. The SWIFT score was calculated using its five constituent parameters: admission source, total ICU length of stay, the last GCS before discharge, and the most recent PaO_2_/FiO_2_ and PaCO_2_ values. To address missingness in physiological data, we employed the forward-filling method to carry over the most recent observations. When arterial blood gas measurements were unavailable, the SpO_2_/FiO_2_ ratio was utilized as a validated clinical proxy for oxygenation status, thereby ensuring cohort representativeness and minimizing patient exclusion.

RNN-based models (RNN, GRU, LSTM, GRU-D, and GRU-D++) were configured with a single hidden layer of 32 units using the tanh activation function to ensure they have similar representational power for a fair comparison. Training employed the binary cross-entropy loss function, with an early stopping criterion if the validation loss did not decrease for 20 consecutive epochs. For XGBoost model, we set the number of boosting rounds to 1000, the learning rate (eta) to 0.01, and the maximum depth to 6. For all other baseline models, we utilized the default hyperparameter settings provided by the scikit-learn library. Regarding the computational environment, all RNN-based models were trained on a single NVIDIA GTX Titan Xp GPU, while the remaining models were executed on an Intel i7-8700 CPU.

## 3. Results

### 3.1. Baseline Characteristics

We used the MIMIC-IV dataset for internal validation and the KNUH dataset for external validation. The MIMIC-IV dataset included 50,261 ICU stays, 46,863 of which resulted in successful discharge and 3398 of which resulted in failure to discharge (i.e., readmitted to the ICU within seven days). The KNUH dataset comprises 4852 ICU stays, of which 4509 were discharged successfully, and 343 were not discharged. Table 1 presents the baseline characteristics of the MIMIC-IV and the KNUH datasets.

### 3.2. Predictive Performances of Machine Learning Models

We developed patient deterioration prediction systems using GRU-D++ and compared them with various artificial intelligence (AI) models. To evaluate these models, we employed two metrics: the area under the receiver operating characteristic curve (AUROC) and the area under the precision-recall curve (AUPR). AUROC is a standard classification metric, whereas the AUPR is particularly suitable for imbalanced data. Table 2 presents the predictive performances of the ML models on the MIMIC-IV and KNUH datasets. For internal validation, we applied ten-fold cross-validation using nine folds for training and one fold for testing while reserving 10% of the training data for model selection. The GRU-D++ model demonstrated the highest performance in internal validation, with an AUROC of 0.802, and achieved an external validation AUROC of 0.756. Additionally, we conducted a fine-tuning experiment to demonstrate that the pre-trained GRU-D++ model (i.e., GRU-D++ trained on MIMIC-IV) can be adapted to a new hospital setting, even with a limited amount of training data. In this experiment, we used 40% of the KNUH data for testing, 10% for model selection, and varying percentages (10%, 20%, 30%, 40%, and 50%) for training. Table 2 presents the results, showing that the predictive performance of the pre-trained GRU-D++ improved as the size of the additional training data increased. In this study, AUROC was employed as the primary metric to ensure standardized benchmarking against existing literature, such as the original SWIFT score, and to evaluate the overall discriminative power of the GRU-D++ architecture across all possible decision thresholds. However, we acknowledge that AUPR is a more sensitive indicator in the context of class imbalance, where the ‘failure’ group represents a small fraction (approximately 7%) of the total population. The observed difference between AUROC and AUPR values reflects the inherent complexity of predicting rare clinical events with high precision. By reporting both metrics, we provide a comprehensive evaluation that balances general model discrimination with the practical challenges of clinical utility in real-world, imbalanced ICU settings.

### 3.3. Comparison with SWIFT

**Figure 3:**
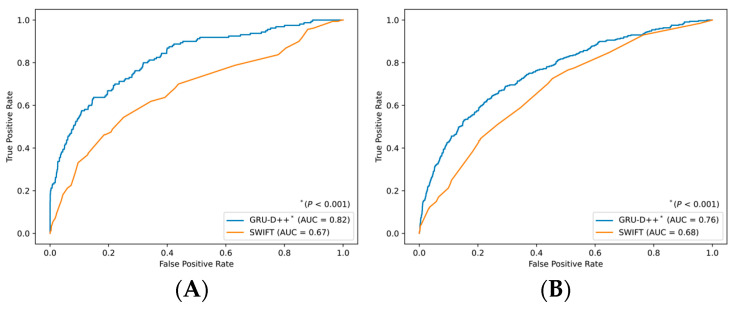
Comparison of predictive performance using GRU-D++ and SWIFT. (**A**) MIMIC-IV and (**B**) KNUH. Abbreviation: AUROC, area under the receiver operating characteristic; SWIFT, stability and Workload Index for Transfer.

The GRU-D++ model was compared with an existing clinical score for ICU readmission, known as the SWIFT score. As shown in Figure 3, GRU-D++ achieved an AUC of 0.82 in the MIMIC dataset, while SWIFT achieved an AUC of 0.69. For the KNUH dataset, GRU-D++ achieved an AUC of 0.76, whereas SWIFT achieved an AUC of 0.68. We performed the DeLong test [23] to compare the statistical significance of AUROCs between SWIFT and GRU-D++. The results showed that GRU-D++ was significantly better than SWIFT (*p* < 0.001). The relatively lower AUROC of the SWIFT score in our study, compared to its original validation, may stem from our use of a composite endpoint that includes post-ICU mortality. Furthermore, consistent with previous external validations, the discriminative power of SWIFT often diminishes when applied to heterogeneous populations with varying institutional discharge practices. Overall, the GRU-D++ model consistently and significantly outperforms the SWIFT model. The clinical significance of our model lies in its ability to refine the triage process during ICU discharge. By providing superior discrimination over the SWIFT score, GRU-D++ can reduce the incidence of unexpected post-discharge complications. For instance, at a fixed specificity, the higher sensitivity of GRU-D++ ensures that more patients prone to 7-day readmission or mortality receive the necessary clinical attention before or immediately after leaving the ICU.

### 3.4. SHapley Value Analysis

**Figure 4:**
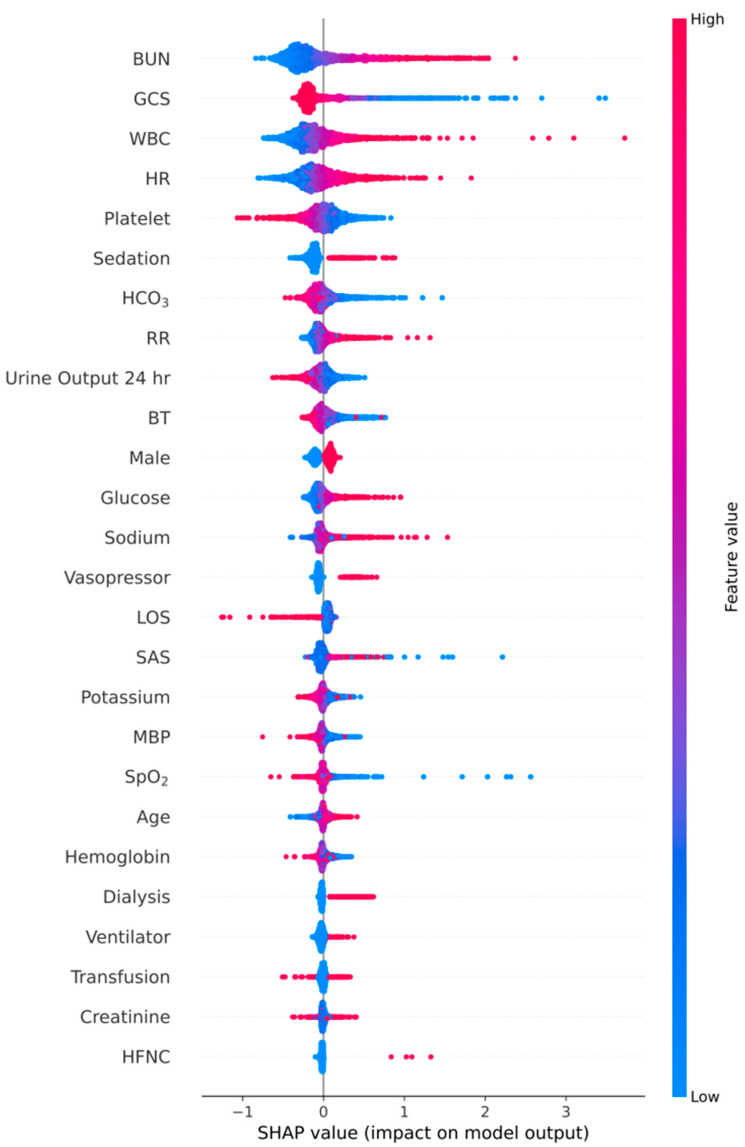
Feature importance and impact on post-discharge deterioration (readmission or death) prediction using Shapley values in ICU patients. The color represents the variable value: red indicates a high value of a specific clinical variable, while blue indicates a low value. Abbreviations: HFNC, high-flow nasal cannula; MBP, mean blood pressure; HR, heart rate; RR, respiratory rate; BT, body temperature; SpO_2_, pulse oxygen saturation; WBC, white blood cell count; BUN, blood urea nitrogen; GCS, Glasgow Coma Scale; SAS, Sedation-agitation scale; LOS, length of stay.

The analysis of SHAP (SHapley Additive exPlanations) [24] values was performed to assess the importance of 26 features concerning the GRU-D++ model (Figure 4). This figure illustrates how high and low feature values are associated with the SHAP values in the dataset. According to the prediction model, a higher SHAP value correlates with an increased probability of readmission and mortality after ICU discharge. In our SHAP analysis, BUN, GCS, and WBC emerged as the most influential features. The strong impact of neurological markers like GCS and SAS underscores the importance of mental status in post-discharge safety. Interestingly, while physiological vitals are common predictors, our model placed high importance on renal markers (BUN and Urine Output), suggesting that metabolic stability is a critical yet sometimes overlooked factor in successful ICU transitions.

### 3.5. Discharge Prediction Scores

**Figure 5:**
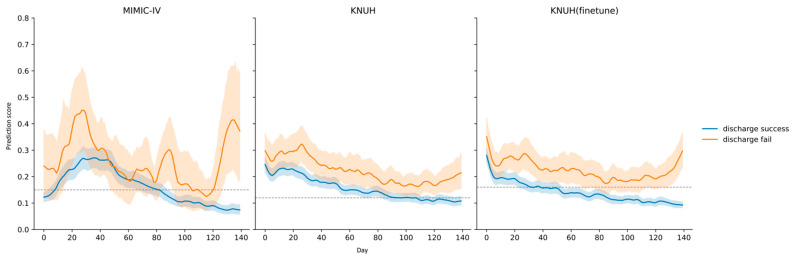
Average prediction scores, where a higher score indicates a higher risk of ICU discharge. The blue line represents the average prediction scores for the discharge success group, and the orange line represents the discharge failure group, both with 95% confidence intervals. The dashed gray line indicates the optimal threshold.

Figure 5 shows the average prediction scores of patients produced by the GRU-D++ model. When analyzing the overall population discharge average prediction score trends using the GRU-D++ model, we observed that the predicted values increased at the point of ICU discharge in the discharge failure group for both the MIMIC-IV and KNUH (fine-tuned) datasets. In contrast, the predicted values decreased in the discharge success group.

**Figure 6:**
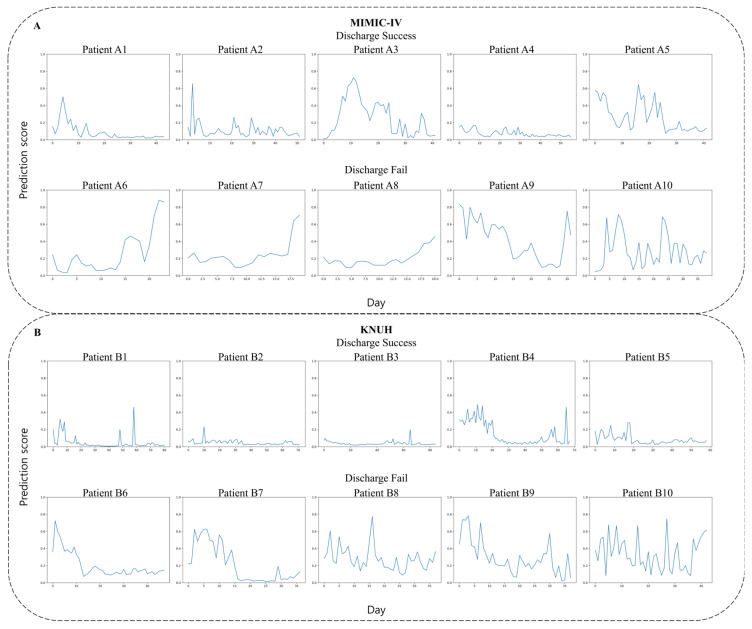
Trends in patients’ discharge prediction scores based on the developed model. (**A**) Random sampling of patients from the MIMIC-IV data. Patient A1–A5: Discharge success group; Patient A6–A10: Discharge failure group. (**B**) Random sampling of patients from the KNUH data. Patient B1–B5: Discharge success group; Patient B6–B10: Discharge failure group.

The risk of post-ICU discharge deterioration was analyzed by randomly extracting five cases from both the discharge success and discharge failure groups for the MIMIC-IV and KNUH datasets. Compared to the discharge failure group, the discharge success group maintained a lower risk. This trend consistently showed low-risk levels up to the time of discharge (Figure 6).

In both the MIMIC-IV and KNUH datasets, the discharge success group shows a consistent trend of decreasing discharge prediction scores from admission to discharge. These groups maintained a stable low discharge prediction score up to the time of discharge, which could clinically facilitate earlier discharge decisions and potentially shorten the length of ICU stay. The individual cases presented in this study reflect the high degree of clinical heterogeneity inherent in ICU populations. While some patients exhibit a clear ‘early warning’ trajectory, others experience more abrupt transitions. The ability of GRU-D++ to maintain predictive performance across these varied patterns—from manifest physiological trends to silent risks—demonstrates its robustness compared to traditional linear assessment tools.

## 4. Discussion

We conducted extensive experiments using various ML and DL models to predict readmission or death within seven days of discharge from the ICU in the MIMIC–IV and KNUH ICU databases. The GRU-D++ model exhibited the best performance for internal validation and achieved the highest AUROC for external validation. GRU-D and GRU-D++ significantly outperformed the other ML and RNN models, demonstrating that the conventional imputation techniques were insufficient for handling missing values. Traditional approaches necessitate manual imputation techniques—such as mean substitution or forward-fill—to address missing data. An additional imputation step prior to training a predictive model requires domain expertise; furthermore, it is difficult to empirically assess the validity of the imputed values. In contrast, GRU-D and GRU-D++ inherently learn internal imputation mechanisms during the training phase. In other words, these models learn optimized imputation mechanisms specifically designed to maximize predictive performance without requiring manual intervention. The trainable imputation methods, GRU-D and GRU-D++, proved to be effective. Furthermore, GRU-D++ outperformed GRU-D, indicating that GRU-D++ was more effective in managing datasets with a higher missing value rate than GRU-D. We evaluated GRU-D++ on the external validation set and determined that it exhibited a good predictive performance (AUROC = 0.756). Additionally, we fine-tuned the pre-trained GRU-D++ using a small amount of additional training data. In this experiment, the performance of GRU-D++ improved steadily as the size of the additional training data set increased. These findings indicate that GRU-D++ can be effectively adopted in a new hospital setting and that its performance improves as more data becomes available.

Previous studies have also reported the results regarding readmission after ICU discharge. Rojas et al. [25] described a machine-learning approach to predict ICU readmission that was significantly more accurate than previously published algorithms, as demonstrated in both internal and external validations for the MIMIC-III cohort. They decided to use a gradient-boosting machine algorithm a priori. They found that their model achieved the highest AUROC score for predicting patients who had ever been readmitted (AUROC = 0.76), followed by SWIFT (AUROC = 0.65) and the Modified Early Warning Score (AUROC = 0.58) for the interval validation cohort. In another study, Pakbin et al. [26] created a model for predicting the risk of ICU readmission at various time points using data from EHRs in the MIMIC-III database. They focused on the last 24 h prior to ICU discharge and employed different classification algorithms, including logistic regression, random forest, and gradient-boosted decision trees. The best result was an AUROC of 0.84 with gradient-boosted decision trees.

Similarly, Xue et al. [27] built graphs of the temporal patterns of variables and then applied frequent subgraph mining. Their experiments were conducted on a more balanced subset of the MIMIC-III, which yielded the best AUROC score of 0.66. Furthermore, Loreto et al. [28] studied the problem of predicting ICU readmissions by using a binary classification task. They tested eight classification algorithms (Bayesian algorithms, decision trees, rule-based methods, and ensemble methods) across different sets of attributes and evaluated their results based on six metrics. Remarkably, their AUROC score of 0.91 (95% CI [0.89, 0.92]) was higher than the existing results published in the literature for other datasets. However, this study highlighted the need for future research utilizing time-series data, as it focused solely on categorical data. In conclusion, while various studies have explored machine learning approaches for predicting ICU readmissions, the need for innovative methods that integrate temporal data and diverse algorithms remains crucial for improving predictive accuracy and clinical outcomes.

In comparison with previous studies, we conducted extensive experiments using various machine learning models to predict patient readmission or death after discharge from the ICU. The experimental results demonstrated that GRU-D++ significantly outperformed conventional machine-learning models, even without missing-value imputation. Unlike traditional models, GRU-D++ does not require a missing-value imputation process, making it highly effective in real-world scenarios. This is particularly important because EHRs often contain numerous missing values that typically need to be imputed using specific methods before being fed into machine-learning models. Consequently, GRU-D++ eliminates this requirement and delivers higher predictive performance than conventional machine-learning models. This robustness was further tested through external validation, where the observed decrease in AUROC (0.802 to 0.756) likely stems from a combination of dataset shifts and institutional variations. Specifically, differences in ethnic backgrounds and baseline physiological parameters between the US-based MIMIC-IV and the Korean KNUH cohorts, alongside divergent clinical protocols regarding discharge criteria and nurse-to-patient ratios, may influence the model’s predictive stability. Additionally, variations in EHR documentation frequencies across different healthcare systems can affect temporal feature extraction. Despite these challenges, the GRU-D++ model’s robust performance (AUROC 0.756) compared to the SWIFT score (AUROC 0.68) in the external cohort suggests it effectively captures universal physiological signatures of deterioration that transcend geographic and systemic boundaries.

## 5. Limitation

This study has several limitations. First, the MIMIC-IV dataset used for model development has been widely utilized in various studies owing to its diverse variables. However, the dataset has a disproportionate representation, with 81.8% White, 10.5% Black, 4.3% Hispanic, and 3.3% Asian populations [29]. Notably, while it may not be appropriate to apply the results of studies conducted solely with MIMIC-IV to Asian populations owing to ethnic distribution, we fine-tuned the KNUH data for this demographic and confirmed that its performance remained stable. To address this demographic and clinical heterogeneity between the MIMIC-IV and KNUH datasets, we utilized standardized inclusion criteria and prioritized universal physiological features. By focusing on objective time-series variables—such as vital signs and laboratory parameters governed by consistent biological principles—we aimed to develop a model that captures fundamental signatures of clinical deterioration regardless of geographic or ethnic background. Although applying results solely from MIMIC-IV to Asian populations may be limited by these baseline differences, our successful fine-tuning and external validation on the ethnically homogeneous KNUH cohort underscores the model’s robustness and its ability to generalize across distinct healthcare systems and diverse case-mixes.

Second, we acknowledge that ICU readmission is fundamentally influenced by systemic and organizational factors beyond individual patient physiology. Factors such as ICU bed occupancy rates, discharge timing (e.g., nighttime or weekend discharges), and the availability of step-down units or rapid response teams play a significant role in determining readmission risk. Due to the retrospective nature of the MIMIC-IV and KNUH datasets, these operational variables were not fully available for inclusion in the current model. Consequently, the model’s predictions should be interpreted primarily as an assessment of physiological stability, and its performance may partly reflect local institutional practices rather than patient status alone. Integrating such systemic proxies remains a vital goal for future work to ensure that AI-driven tools align more closely with real-world clinical environments.

Third, the ICU discharge failure group included patients with a “do not resuscitate” status who may choose to be discharged despite anticipated deterioration by refusing additional invasive treatments. As a result, in the analysis of the MIMIC-IV data, it was difficult to identify patients with this status; therefore, they were included in the development dataset. Consequently, identifying the specific impact of DNR status on predictive accuracy remains a limitation. Future research should prioritize the integration of DNR and palliative care data to better distinguish between preventable deterioration and expected clinical outcomes, thereby enhancing the model’s practical utility in end-of-life care transitions.

Fourth, this study included all the disease categories of patients admitted to the ICU, demonstrating the heterogeneity of the study population. As a result, predicting the discharge timing using the same system for heterogeneous groups can be challenging. A further limitation of this study is the use of a composite endpoint, which combines ICU readmission and post-ICU death. Since these two outcomes often represent distinct clinical trajectories—where readmission may be potentially preventable through enhanced monitoring while mortality may involve different physiological declines—they warrant separate analysis. Future studies with larger cohorts should evaluate these outcomes independently to refine the model’s clinical utility and provide more granular insights into specific patient trajectories.

Fifth, the class imbalance (approximately 7% failure rate) in both datasets may affect the model’s precision; however, the use of AUPRC and comparison with established scores suggest clinical relevance.

Sixth, while GRU-D++ is robust in handling missing data patterns (MAR/MNAR) common in ICUs, the potential for temporal drift in historical data remains a limitation. Furthermore, this study primarily focused on performance at the time of discharge. Future research should investigate the lead time for early warnings and establish optimal decision thresholds through Decision Curve Analysis (DCA) to facilitate practical clinical implementation.

It is also important to consider that patients in real-world ICU settings often present with multiple complex conditions rather than a single disease. Therefore, this comprehensive evaluation approach may be advantageous in real-world applications. Such limitations could serve as an advantage for models, such as GRU-D++, which are capable of processing missing data. This model is expected to be clinically useful in the real-world setting. Additionally, as shown in Figure 5, the GRU-D++ model was used to assess the ICU discharge risk in time-series data and to compare trends between the discharge success and failure groups, thereby suggesting its feasibility for future use in clinical practice. Ultimately, the GRU-D++ model shows potential as a tool that can assist in decision-making, thereby reducing the workload of physicians. This model aids in early interventions for patients at risk of deterioration and supports the discharge of stable patients.

Our study was conducted retrospectively, and additional research in a large-scale, multi-institutional setting is necessary to enhance its generalizability. Furthermore, well-designed prospective clinical trials are required to demonstrate the effectiveness of the GRU-D++ model as a screening tool in clinical practice. While our current focus was on developing a highly generalizable model for diverse ICU populations, we acknowledge that different disease categories exhibit distinct clinical nuances. Therefore, future research will prioritize detailed subgroup analyses to refine predictive precision for specific pathologies, facilitating the implementation of a more sophisticated and specialized hospital-wide decision-support tool.

To facilitate clinical implementation, the GRU-D++ model is designed for integration into Electronic Health Record (EHR) systems to provide continuous risk trajectories during the 24–48 h discharge decision window. High-risk predictions, accompanied by explainable SHAP-based factors, would be communicated via a clinical decision support dashboard to trigger a ‘Safety Pause’ or prioritize patients for step-down units and rapid response team follow-ups. The primary benefit of this system lies in its ability to optimize ICU throughput by reducing the incidence of preventable readmissions and associated clinical complications. By providing objective decision-support, the model minimizes the clinical uncertainty that often leads to prolonged ICU stays or premature discharges, thereby fostering a more efficient allocation of specialized medical resources and ensuring the long-term sustainability of critical care services.

## 6. Conclusions

Physicians face a challenge in determining which patients are ready for ICU discharge, and there is a lack of tools to assist them in making this decision. Overall, our findings suggest that the GRU-D++ model has the potential to assist clinicians in more accurately identifying high-risk patients who are at risk of readmission or death after ICU discharge, ultimately leading to timely interventions and shorter ICU stays. To facilitate clinical implementation, the GRU-D++ model is designed for seamless integration into Electronic Health Record (EHR) systems, providing continuous risk trajectories during the critical 24–48 h discharge decision window. In practice, high-risk predictions—supported by SHAP-based explainable factors—will be communicated via centralized dashboards to trigger a ‘Safety Pause’ or prioritize patients for step-down units and rapid response team follow-ups. Future research should prioritize prospective multi-center ‘shadow studies’ to calibrate decision thresholds and address implementation barriers, such as diverse institutional protocols and DNR cases. Ultimately, this proactive risk stratification framework will bridge the gap between model development and bedside application, enhancing patient safety while securing financial sustainability by optimizing the allocation of medical resources in high-pressure ICU environments.

## Figures and Tables

**Table 1:** Baseline characteristics 24 h before ICU discharge.

	MIMIC-IV					KNUH				
	All (*N* = 50,261)	Discharge Success (*N* = 46,863)	Discharge Failed (*N* = 3398)	*p* Value	Missing Value	All (*N* = 4852)	Discharge Success (*N* = 4509)	Discharge Failed (*N* = 343)	*p* Value	Missing Value
Sex, Male, *n* (%)	28,377 (56.46%)	26,529 (56.61%)	1848 (54.38%)	0.012	0.00%	2881 (59.38%)	2676 (59.35%)	205 (59.77%)	0.909	0.00%
Age (years), median (IQR)	66.83 (55.21–77.58)	66.46 (54.89–77.21)	72.05 (60–82.3)	<0.001	0.00%	74 (61–82)	74 (60–81)	77 (64.5–83)	<0.001	0.00%
**Procedures and therapies**										
HFNC, *n* (%)	209 (0.42%)	166 (0.35%)	43 (1.27%)	<0.001	4.18%	259 (5.34%)	201 (4.46%)	58 (16.91%)	<0.001	0.00%
Ventilator, *n* (%)	2588 (5.15%)	2406 (5.13%)	182 (5.36%)	0.574	2.90%	148 (3.05%)	107 (2.37%)	41 (11.95%)	<0.001	0.00%
Dialysis, *n* (%)	1728 (3.44%)	1509 (3.22%)	219 (6.44%)	<0.001	2.86%	34 (0.70%)	25 (0.55%)	9 (2.62%)	<0.001	0.00%
Vasopressor, *n* (%)	1027 (2.04%)	899 (1.92%)	128 (3.77%)	<0.001	2.33%	99 (2.04%)	81 (1.80%)	18 (5.25%)	<0.001	0.00%
Sedation, *n* (%)	2843 (5.66%)	2468 (5.27%)	375 (11.04%)	<0.001	2.75%	248 (5.11%)	229 (5.08%)	19 (5.54%)	0.702	0.00%
Transfusion, *n* (%)	2398 (4.77%)	2234 (4.77%)	164 (4.83%)	0.868	9.95%	116 (2.39%)	99 (2.20%)	17 (4.96%)	0.005	0.00%
**Physiologic parameters**										
MBP (mmHg), median (IQR)	82.69 (75.33–91.41)	82.77 (75.48–91.44)	81.15 (73.09–91)	<0.001	5.11%	88.71 (81.21–96.75)	88.75 (81.35–96.73)	88.47 (79.84–96.88)	0.445	1.57%
HR (pulse/min), median (IQR)	81.79 (72.2–91.88)	81.44 (72–91.33)	87.85 (76.13–99.33)	<0.001	5.89%	82.07 (71.46–93.67)	81.3 (71.2–92.86)	92.71 (81.61–103.55)	<0.001	0.95%
RR (insp/min), median (IQR)	18.77 (16.55–21.38)	18.7 (16.53–21.25)	20 (17–23.06)	<0.001	5.50%	19.25 (17.11–21.69)	19.12 (17–21.45)	21.64 (18.87–24.41)	<0.001	0.95%
BT (°C), median (IQR)	36.81 (36.63–37.01)	36.81 (36.63–37.01)	36.76 (36.53–37)	<0.001	5.58%	36.78 (36.53–37.06)	36.78 (36.54–37.06)	36.72 (36.44–37.11)	0.027	1.07%
SpO_2_ (%), median (IQR)	96.33 (95.09–97.58)	96.35 (95.14–97.59)	96.06 (94.32–97.49)	<0.001	5.15%	99.29 (97.96–99.88)	99.29 (98–99.89)	99.08 (97.7–99.78)	0.002	0.95%
Urine Output 24 h (mL), median (IQR)	1575 (960–2400.75)	1600 (980–2425)	1272 (715–2085)	<0.001	5.08%	1090 (705–1575)	1100 (720–1600)	900 (565–1310)	<0.001	3.69%
**Laboratory findings**										
HCO_3_ (mEq/L), median (IQR)	25 (23–28)	25 (23–28)	25 (22–28)	<0.001	5.02%	21.4 (18.9–24.1)	21.3 (18.9–24.1)	22.2 (18.95–25.3)	0.032	38.27%
WBC (×10^3^/µL), median (IQR)	9.4 (7.1–12.5)	9.4 (7–12.4)	10.2 (7.45–13.9)	<0.001	4.91%	8.8 (6.6–11.53)	8.7 (6.5–11.3)	10.5 (7.8–15.4)	<0.001	16.98%
Hemoglobin (g/dL), median (IQR)	10 (8.75–11.4)	10 (8.8–11.5)	9.4 (8.3–10.8)	<0.001	4.87%	10.3 (9–11.8)	10.3 (9.1–11.8)	9.9 (8.8–11.1)	<0.001	16.88%
Platelet (×10^3^/µL), median (IQR)	189 (135–259)	189 (136–259)	186 (118–263)	<0.001	0.00%	178 (120–247)	178 (121–247)	179 (107.25–251.75)	0.583	16.88%
Glucose (mg/dL), median (IQR)	115 (99–139)	115 (99–138)	123 (101–150)	<0.001	0.00%	121 (103.75–152)	120.37 (103.5–151)	129 (107.58–162.88)	0.001	2.51%
BUN (mg/dL), median (IQR)	18 (12–29.5)	18 (12–28.5)	27 (17–46)	<0.001	0.00%	17 (11.5–27.58)	16.5 (11.3–26.9)	23 (15.35–40.85)	<0.001	17.19%
Creatinine (mg/dL), median (IQR)	0.9 (0.7–1.3)	0.9 (0.7–1.3)	1.1 (0.7–1.8)	<0.001	0.00%	0.7 (0.5–1.1)	0.7 (0.5–1.1)	0.8 (0.5–1.61)	0.014	17.21%
Sodium (mEq/L), median (IQR)	138.5 (136–141)	138.5 (136–141)	139 (136–143)	<0.001	0.00%	139 (136–141)	139 (136–141)	138 (134.5–141.13)	0.036	12.26%
Potassium (mEq/L), median (IQR)	4 (3.7–4.3)	4 (3.7–4.3)	4 (3.7–4.35)	0.241	0.00%	3.8 (3.49–4.11)	3.8 (3.48–4.1)	3.85 (3.5–4.22)	0.028	12.26%
GCS, median (IQR)	15 (14.6–15)	15 (14.67–15)	14.67 (13.5–15)	<0.001	4.83%	14.58 (14–15)	14.71 (14–15)	14 (11–15)	<0.001	1.50%
SAS, median (IQR)	4 (4–4)	4 (4–4)	4 (4–4)	<0.001	8.38%	4 (4–4)	4 (4–4)	4 (3–4)	<0.001	20.65%
LOS (days), median (IQR)	2.28 (1.51–4.11)	2.24 (1.49–4.01)	3.15 (1.9–6.04)	<0.001	0.00%	3 (2–6)	3 (2–6)	5 (3–11)	<0.001	0.00%

Data are presented as numbers with percentages (*n*, %) or median (IQR). Values were calculated between the discharge success and discharge failure groups using fisher’s exact test for binary variables or Mann–Whitney U tests for numerical variables, as appropriate. Statistical significance was set at *p* < 0.05. Abbreviation: HFNC, high-flow nasal cannula; MBP, mean blood pressure; HR, heart rate; RR, respiratory rate; BT, body temperature; SpO_2_, saturation pulse oxygen; WBC, white blood cell; BUN, blood urea nitrogen; GCS, Glasgow Coma Scale; SAS, sedation-agitation scale; LOS, length of stay in the ICU.

**Table 2:** Predictive performance comparison of models using MIMIC-IV and KNUH data. Values represent mean (±std) over 10-fold cross-validation.

Model	MIMIC-IV		KNUH	
	AUROC	AUPR	AUROC	AUPR
LR	0.769 (±0.012)	0.317 (±0.018)	0.759 (±0.001)	0.260 (±0.002)
DT	0.715 (±0.014)	0.226 (±0.024)	0.665 (±0.016)	0.160 (±0.011)
RF	0.779 (±0.014)	0.343 (±0.016)	0.736 (±0.007)	0.237 (±0.006)
XGB	0.777 (±0.014)	0.331 (±0.020)	0.736 (±0.003)	0.230 (±0.004)
RNN	0.784 (±0.016)	0.346 (±0.021)	0.746 (±0.005)	0.247 (±0.006)
GRU	0.787 (±0.015)	0.352 (±0.021)	0.745 (±0.005)	0.242 (±0.008)
LSTM	0.787 (±0.015)	0.355 (±0.019)	0.747 (±0.011)	0.240 (±0.012)
GRU-D	0.799 (±0.011)	0.382 (±0.019)	0.757 (±0.005)	0.249 (±0.010)
GRU-D++	0.802 (±0.010)	0.383 (±0.020)	0.756 (±0.005)	0.237 (±0.010)
GRU-D++ (fine-tuned 10%)	-	-	0.752 (±0.009)	0.243 (±0.014)
GRU-D++ (fine-tuned 20%)	-	-	0.755 (±0.010)	0.250 (±0.013)
GRU-D++ (fine-tuned 30%)	-	-	0.759 (±0.012)	0.249 (±0.016)
GRU-D++ (fine-tuned 40%)	-	-	0.761 (±0.012)	0.251 (±0.014)
GRU-D++ (fine-tuned 50%)	-	-	0.765 (±0.012)	0.258 (±0.015)

Abbreviation: LR, Logistic regression; DT, Decision tree; RF, Random forest; XGB, eXtreme Gradient Boosting; RNN, Recurrent Neural Network; GRU, Gated Recurrent Unit; LSTM, Long Short-Term Memory.

## Data Availability

The data that support the findings of this study are available on request from the corresponding author. The data are not publicly available due to privacy or ethical restrictions.

## Supplementary Materials

The following supporting information can be downloaded at: https://www.mdpi.com/article/10.3390/diagnostics16060874/s1, Table S1. The initial variables and the missing rate for each variable; Figure S1. Normal distribution graphs of MIMIC variables with *p* < 0.001. (a) Age, (b) MBP, (c) HR, (d) RR, (e) BT, (f) SpO_2_, (g) Urine Output 24 h, (h) HCO_3_, (i) WBC, (j) Hemoglobin, (k) Platelet, (l) Glucose, (m) BUN, (n) Creatinine, (o) Sodium, (p) Potassium, (q) GCS, (r) SAS, and (s) LOS; Figure S2. Normal distribution graphs of KNUH variables with *p* < 0.001. (a) Age, (b) MBP, (c) HR, (d) RR, (e) BT, (f) SpO_2_, (g) Urine Output 24 h, (h) HCO_3_, (i) WBC, (j) Hemoglobin, (k) Platelet, (l) Glucose, (m) BUN, (n) Creatinine, (o) Sodium, (p) Potassium, (q) GCS, (r) SAS, and (s) LOS.

## Abbreviations

The following abbreviations are used in this manuscript:

ICU	Intensive care unit
MIMIC-IV	Medical Information Mart for Intensive Care IV
KNUH	Kangwon National University Hospital
AUROC	Area under the receiver operating characteristic
SWIFT	Stability and Workload Index for Transfer
APACHE	Acute Physiology and Chronic Health Evaluation
GCS	Glasgow Coma Scale
ED	Emergency department
LR	Logistic regression
DT	Decision tree
RF	Random forest
XGB	eXtreme Gradient Boosting
GRU	Gated recurrent unit
RNN	Recurrent neural network
LSTM	Long short-term memory
HFNC	High-flow nasal cannula
MBP	Mean blood pressure
HR	Heart rate
RR	Respiratory rate
BT	Body temperature
SpO_2_	Saturation pulse oxygen
WBC	White blood cell count
BUN	Blood urea nitrogen
SAS	Sedation-Agitation Scale
LOS	Length of stay
AUPR	Area under the precision-recall curve
SHAP	SHapley additive exPlanations

## Appendix A. GRU-D++

GRU-D++ was used as the main predictive model in this study. The primary advantage of GRU-D++ is that it automatically imputes missing values without human effort. Let

$$X=\left(x_{1},x_{2},\ldots ,x_{T}\right)\in R^{T\times M}$$

Be a multivariate time series, where T is the number of time steps, and M is the number of input variables. Given **X**, GRU-D++ defines the missing-value mask

$${M\in \{0,1\}}^{T\times M}\;\mathrm{for}\;X\;\mathrm{as}$$

$$\mu_{t,m}=\left\{\begin{array}{c} 0,\;if\;x_{t,m}\;is\;N/A\;\\ 1,\;otherwise \end{array}\in M\right.$$

where N/A indicates missing values, and the last observation value $\tilde{X}$ of the input variables and corresponding missing value mask $\tilde{M}$ for $\tilde{X}$ are defined as follows:

$${\tilde{x}}_{t,m}=\left\{\begin{array}{c} x_{t,m},\;if\;\mu_{t,m}=1\\ {\tilde{x}}_{t-1,m},\;if\;\mu_{t,m}=0\;and\;t>1\in \tilde{X}\\ N/A \end{array}\right.$$

$${\tilde{\mu }}_{t,m}=\left\{\begin{array}{c} 0,\;if\;\;{\tilde{x}}_{t,m}\;is\;N/A\\ 1,\;othterwise \end{array}\;\in M\right.$$

We recorded the intervals between time steps $∆\in R^{T\times M}$ as follows:

$$\delta_{t,m}=\left\{\begin{array}{c} x_{t,m}-s_{t-1}+\delta_{t-1,m},\;if\;t>1\;and\;\mu_{t-1,m}=0\\ x_{t,m}-s_{t-1},\;if\;t>1\;and\;\mu_{t-1,m}=1\in \tilde{X}\\ 0,\;otherwise \end{array}\right.$$

where $s=\left(s_{1},s_{2},\ldots ,s_{T}\right)\in \;R^{T}$ represents the timestamp of x. Because the time intervals between observations can differ, we applied trainable decay mechanisms to the input variables and hidden states of GRU-D++. The decaying weights $\gamma_{x_{t}}$ and $\gamma_{h_{t}}$ of the input variables and hidden states are defined as follows:

$$\gamma_{x_{t}}=\;\exp\left(-\max\left(0,\;\delta_{t}W_{x_{t}}+\;b_{x_{t}}\right)\right)⨀{\tilde{\mu }}_{t},$$

$$\gamma_{h_{t}}=\;\exp\left(-\max\left(0,\;\delta_{t}W_{h_{t}}+{\;b}_{h_{t}}\right)\right),$$

where $W_{x_{t}}\in R^{M\times M}$, $W_{h_{t}}\in R^{M\times K}$, $b_{x_{t}}\in R^{M}$, and $b_{h_{t}}\in R^{K}$ are the learnable parameters. The final imputation mechanism is defined as follows:

$$x_{t,m}^{\prime }=m_{t,m}x_{t,m}+(1-m_{t,m})\times (\gamma_{x_{t},m}{\tilde{x}}_{t,m}+(1-\gamma_{t,m}){\bar{x}}_{m})$$

where ${\bar{x}}_{m}$ is the arithmetic mean of the mth variable. GRU-D++ is defined as follows:

$$h_{t-1}^{\prime }={\;\gamma }_{h_{t}}h_{t-1},$$

$$r_{t}=\sigma \left(x_{t}^{\prime }W_{r}+h_{t-1}^{\prime }U_{r}+m_{t}V_{r}+{\tilde{m}}_{t}Q_{r}+b_{r}\right),$$

$$z_{t}=\sigma \left(x_{t}^{\prime }W_{z}+h_{t-1}^{\prime }U_{z}+m_{t}V_{z}+{\tilde{m}}_{t}Q_{z}+b_{z}\right),$$

$${\tilde{h}}_{t}=tanh\left(x_{t}^{\prime }W+(r_{t}⨀h_{t-1}^{\prime })U+m_{t}V+\tilde{m}Q+b\right),$$

$$h_{t}=\left(1-z_{t}\right)⊙h_{t-1}^{\prime }+z_{t}⊙{\tilde{h}}_{t}$$
