# Supplementary material for: AI-Driven Predictions of Readmission and Mortality for Improved Discharge Decisions in Critical Care: A Retrospective Study
Source: Diagnostics (Basel). 2026 Mar 16;16(6):874. doi: 10.3390/diagnostics16060874 (PMC13025911; doi:10.3390/diagnostics16060874)
Supplement: Supplementary file 1 [file diagnostics-16-00874-s001.zip › diagnostics-4150568-supplementary.pdf]

diagnostics

MDPI

Supplementary Table

We used 26 variables with less than 50% missing data among the initial 48 candidate variables for the final analysis. The full list of the 48 initial variables and the proportion of missing values in each dataset are provided in Table S1.

Table S1. The initial variables and the missing rate for each variable.

Variables	Missing rate
Sex	0.000%
Age	0.000%
**Procedures and therapies**	
HFNC	0.000%
Ventilation	0.000%
Dialysis	0.000%
Antibiotic	0.000%
Vasopressor	0.000%
Sedation	0.000%
Transfusion	0.000%
**Physiologic parameters**	
DBP	1.532%
HR	1.296%
RR	1.547%
BT	3.849%
SpO_2	1.445%
Urine Output 24hr	8.554%
BMI	90.165%
**Underlying diseases**	
Cerebrovascular disease	0.000%
Diabetes	0.000%
Hypertension	0.000%
Asthma	0.000%
COPD	0.000%
Chronic kidney disease	0.000%
Heart failure	0.000%
Cancer	0.000%
Cirrhosis	0.000%
**Laboratory findings**	
pH	55.421%
pCO_2	60.431%
pO_2	60.422%
HCO_3	7.307%
WBC	8.321%
Hemoglobin	8.023%
Platelet	8.258%
Lactate	67.671%
CRP	99.061%
Glucose	7.018%
Albumin	84.139%
Total bilirubin	70.822%
AST	70.498%

ALT	70.721%
ALP	71.066%
BUN	7.270%
Creatinine	7.216%
Sodium	6.768%
Potassium	6.681%
Calcium	13.584%
GCS	2.571%
SAS	4.776%
LOS	0.000%

Abbreviations: HFNC, High flow nasal cannula; DBP, Diastolic blood pressure; SBP, Systolic blood pressure; HR, Heart rate; RR, Respiratory rate; BT, Body temperature; BMI, Body mass index; COPD, Chronic obstructive pulmonary disease; WBC, White blood cell; CRP, C-reactive protein; AST, Aspartate aminotransferase; ALT, Alanine transaminase; ALP, Alkaline phosphatase; BUN, Blood Urea Nitrogen; GCS, Glasgow Coma Scale; SAS, Sedation-Agitation Scale;.

Supplementary Figure

As shown in Figures S1 and S2, we examined the normality of 19 variables with **P** < 0.001. Figure S1 shows the distributions for the MIMIC variables, whereas Figure S2 shows those for the KNUH variables.

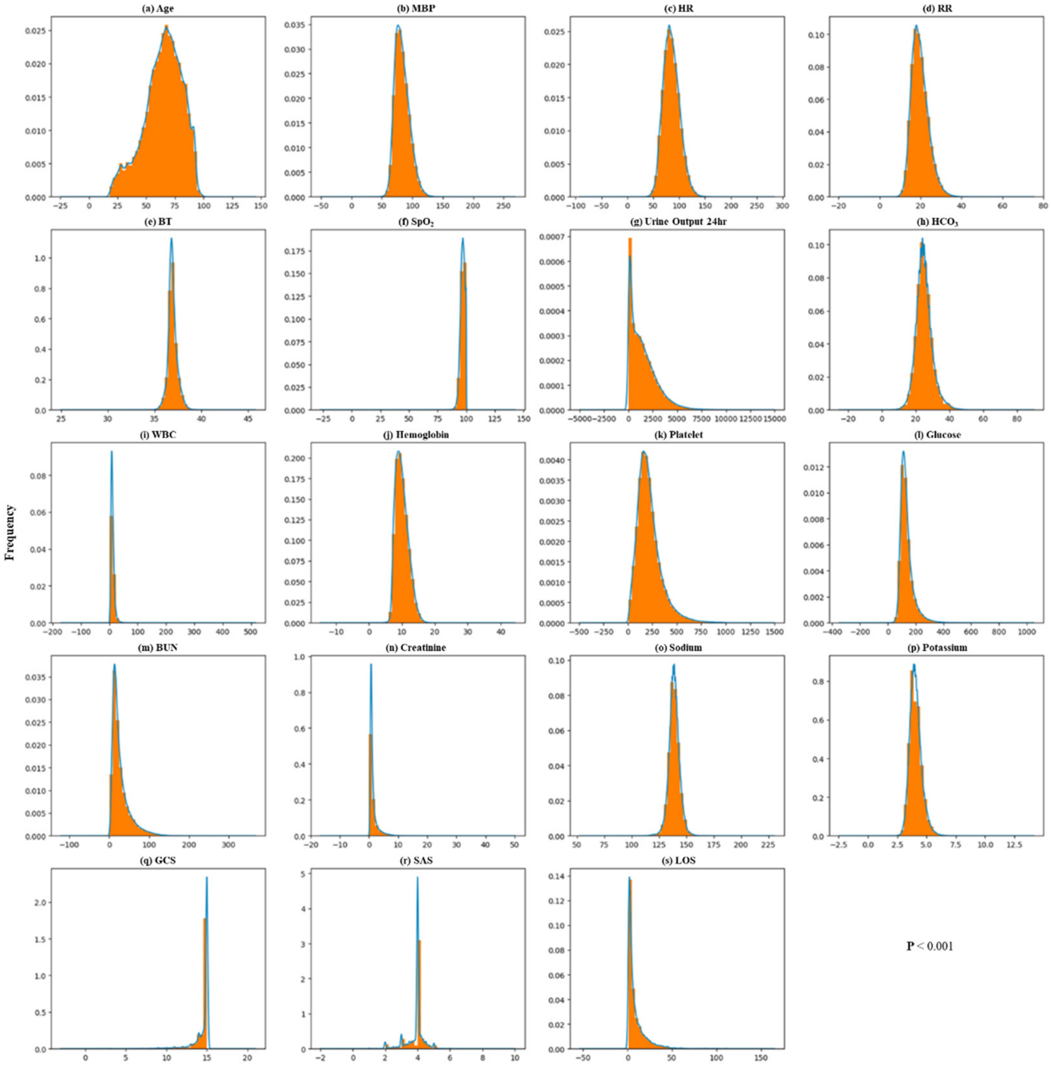

Fig. S1 Normal distribution graphs of MIMIC variables with **P** < 0.001. (a) Age, (b) MBP, (c) HR, (d) RR, (e) BT, (f) SpO_2, (g) Urine Output 24hr, (h) HCO_3, (i) WBC, (j) Hemoglobin, (k) Platelet, (l) Glucose, (m) BUN, (n) Creatinine, (o) Sodium, (p) Potassium, (q) GCS, (r) SAS, and (s) LOS

Abbreviations: MBP, Mean blood pressure; HR, Heart rate; RR, Respiratory rate; BT, Body temperature; WBC, White blood cell; BUN, Blood Urea Nitrogen; GCS, Glasgow Coma Scale; SAS, Sedation-Agitation Scale; LOS, Length of stay;

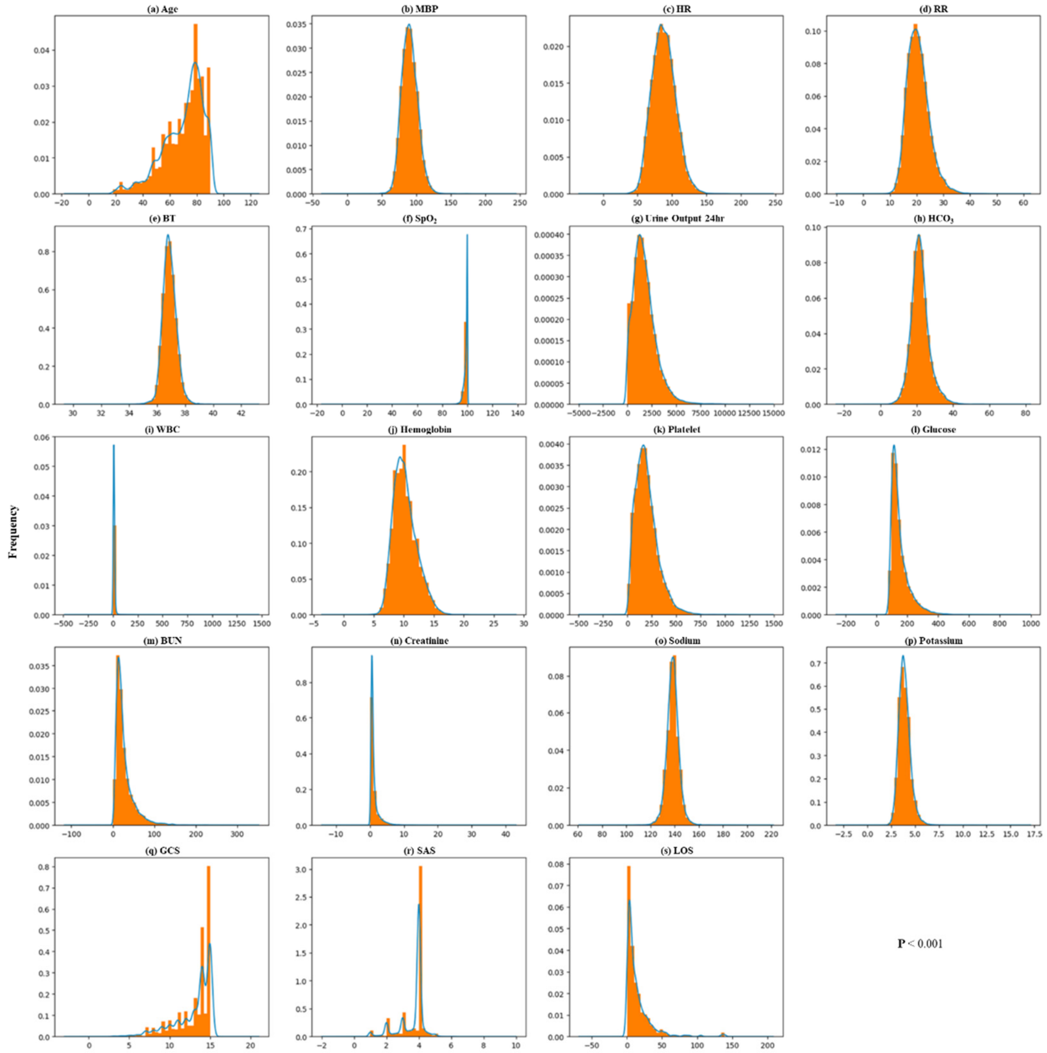

Fig. S2 Normal distribution graphs of KNUH variables with **P** < 0.001. (a) Age, (b) MBP, (c) HR, (d) RR, (e) BT, (f) SpO_2, (g) Urine Output 24hr, (h) HCO_3, (i) WBC, (j) Hemoglobin, (k) Platelet, (l) Glucose, (m) BUN, (n) Creatinine, (o) Sodium, (p) Potassium, (q) GCS, (r) SAS, and (s) LOS

Abbreviations: MBP, Mean blood pressure; HR, Heart rate; RR, Respiratory rate; BT, Body temperature; WBC, White blood cell; BUN, Blood Urea Nitrogen; GCS, Glasgow Coma Scale; SAS, Sedation-Agitation Scale; LOS, Length of stay
